# Dynamic 2-deoxy-2[18F] fluoro-D-glucose PET/MRI in human renal allotransplant patients undergoing acute kidney injury

**DOI:** 10.1038/s41598-020-65267-8

**Published:** 2020-05-19

**Authors:** Sahra Pajenda, Sazan Rasul, Marcus Hacker, Ludwig Wagner, Barbara Katharina Geist

**Affiliations:** 1 Department of Medicine III, Division of Nephrology and Dialysis, Medical University of Vienna. Waehringer Guertel 18-20, 1090 Vienna, Austria; 2 Department of Biomedical Imaging and Image- Guided Therapy, Division of Nuclear Medicine, Medical University of Vienna. Waehringer Guertel 18-20, 1090 Vienna, Austria

**Keywords:** Medical research, Nephrology, Medical imaging

## Abstract

Patients after solid organ kidney transplantation (KTX) often suffer from acute kidney injury (AKI). Parameters as serum creatinine indicate a loss of kidney function, although no distinction of the cause and prognosis can be made. Imaging tools measuring kidney function have not been widely in clinical use. In this observational study we evaluated 2-deoxy-2[18F] fluoro-D-glucose (FDG) PET/MRI in thirteen patients after KTX with AKI as a functional assessment of the graft. Twenty-four healthy volunteers served as control. General kidney performance (GKP), initial flow (IF) and renal response function (RF) were calculated by standardized uptake values (SUV) and time activity curves (TAC). The GKP measured for the total kidney and medulla was significantly higher in healthy patients compared to patients after KTX (p = 0.0002 and p = 0.0004, respectively), but no difference was found for the GKP of the cortex (p = 0.59). The IF in KTX patients correlated with renal recovery, defined as change in serum creatinine 10 days after PET/MRI (r = 0.80, p = 0.001). With regard to the RF, a negative correlation for tubular damage was found (r = −0.74, p = 0.004). In conclusion, parameters obtained from FDG PET/MRI showed a possible predictive feature for renal recovery in KTX patients undergoing AKI.

## Introduction

Within the first year after kidney transplantation (KTX) postoperative monitoring and care is crucial for patients’ outcome. Delayed graft function (DGF) and early rejection representing a subtype of acute kidney injury (AKI) are common and make up for 20–40% of cases^1–4^. The most susceptible site to injury by oxygen and energy deprivation is the epithelia of the proximal tubule^5^. Tubular cell injury is inflicted by hours of cold ischemia time during transportation from donor to recipient site^6,7^. Among other potential factors for kidney dysfunction^8^ is the kidney donor profile index (KDPI)^9^ which most likely influences the organ-specific regenerative potential^10–12^. Furthermore acceptance of expanded criteria donor (ECD) and donation after circulatory death (DCD) with prolonged warm ischemia time have led to increasing incidence of DGF^13–15^. Potential nephrotoxic medication as calcineurin inhibitors required after kidney transplantation is an additional risk factor for acute kidney injury^16^.

Renal tubular cells are capable of replicating and can repopulate injured regions. This reflects the organ specific regenerative potential and is variable from individual to individual^17–19^. Therefore renal recovery following transplantation or kidney injury due to acute events differs markedly and no reliable parameters are available in predicting the time span of allograft function regain^20,21^.

In case of kidney function deterioration, a core needle biopsy is inevitable to confirm the diagnosis and determine the further course. One essential focus is the establishment of diagnostic tools to identify the underlying cause of renal dysfunction and the determination of predictive markers for the restoration of renal function. Various biomarkers have been tested for their diagnostic value for partial or full organ recovery following AKI in transplant recipients^22–25^. Markers such as KIM-1, IGFBP7, TIMP-2 and other have shown promising results in predicting delayed graft function prior to known laboratory tests^26^. However, too many factors and pathophysiological states represent the underlying causes of AKI that not one single biomarker in blood or urine could provide sufficient information for the clinician in directing the care procedures^27–30^.

Non- invasive radio imaging such as sonography is a further important surveillance tool in allograft assessment^31,32^. Although most of the imaging methods are easily accessible only estimates can be provided in respect to graft structure and perfusion, but not on complex renal function^33–35^. Computed tomography (CT) on one side provides higher resolution but on the other side radio contrast should be better avoided in the early stages of transplantation^31,36^. Despite all this, core needle biopsy still remains the gold standard in detecting the reason of malfunction.

In the past decade novel technology has provided additional imaging tools by combining positron emission tomography (PET) with CT or magnetic resonance imaging (MRI) using the glucose analogue radio tracer 2-deoxy-2[18F] fluoro-D-glucose (FDG)^37^. PET/MRI scans give information on both, accurate organ substructures and functional components^38^. Additionally, with dynamic scans the behavior of the tracer can be observed in the kidneys, undergoing several renal processes such as filtration, re-absorption and excretion^39^.

In this observational study, we analyzed the functional behavior of the glucose analogue FDG by the use of novel PET/MRI technology in patients after kidney transplantation with AKI. Kidney functional parameters were compared with those from healthy volunteers, which were acquired in a previous study^40,41^. Using the MRI sequences to determine the localization of the renal cortex and medulla combined with the dynamic PET images, information on regional cellular functionality was obtained.

In parallel, we followed the function of the excretory kidney with parameters such as serum creatinine and additionally compared the PET/MRI results with renal biopsies taken close to the time of examination.

## Methods

The study was carried out at the Division of Nephrology and Dialysis in Collaboration with the Division of Nuclear Medicine at the Medical University of Vienna between 2016 and 2019. Thirteen kidney recipients admitted to hospital due to DGF or kidney function deterioration were enrolled in the study. Demographic data and routine parameters were extracted from the data base of the General Hospital of Vienna. Inclusion criteria were age above 18 years, kidney transplantation regardless the immunological risk constellation, suitable to undergo PET/MRI. Exclusion criteria were claustrophobia, existing metal prosthesis and implantation of any metal devices.

All patients were monitored for their renal function by routine blood and urine testing and gave written and oral consent. The study was approved by the Local Ethics Committee of the Medical University of Vienna (protocol: 1043/2016). The study was conducted in accordance to the Declaration of Helsinki and relevant guidelines and regulations and no organs/ tissues were procured from prisoners.

### Kidney biopsy

Tissues from fine needle biopsies were immediately fixed by Formalin and embedded in paraffin. Histological processing and diagnosis was performed at the Department of Pathology at the Medical University of Vienna according to the BANFF classification. Additional immunohistological staining was carried out for presence of C4d for evaluating antibody mediated rejection (ABMR).

### Recovery determination

On a daily base, blood samples were drawn from all patients before and after PET/MRI examinations, from which serum creatinine was determined and eGFR was calculated according to CKD-EPI formula^42^.

A positive recovery was defined when serum creatinine decreased by >10% ten days after the PET/MRI examination, a negative recovery when serum creatinine increased by >10% ten days after the PET/MRI examination and an unchanged status was classified when the change was between ± 10%.

### PET/MRI examination protocol

Directly before scan start, around 3 MBq/kg body weight FDG (isotope: fluoride-18, half-life: 109.7 min) were injected. The PET/MRI (Siemens Biograph mMR, Siemens Healthcare Diagnostics GmbH, Germany) acquisition started immediately after tracer injection and continued for 24 minutes, PET data sets were reconstructed (Siemens e7 tools) into a dynamic sequence of 60 × 5 s, 19 ×60 s a 172 × 172 × 127 matrix using the ordinary Poisson ordered subsect expectation maximization (OP-OSEM) 3D algorithm (3 iterations, 21 subsets, Gaussian filter). Scatter correction along with Dixon based MR-attenuation correction was performed. The MR imaging protocol consisted of a T1 weighted MRI sequence (axial breath holding and fat suppression, vibe spair).

### Image data analysis

**Figure 1:**
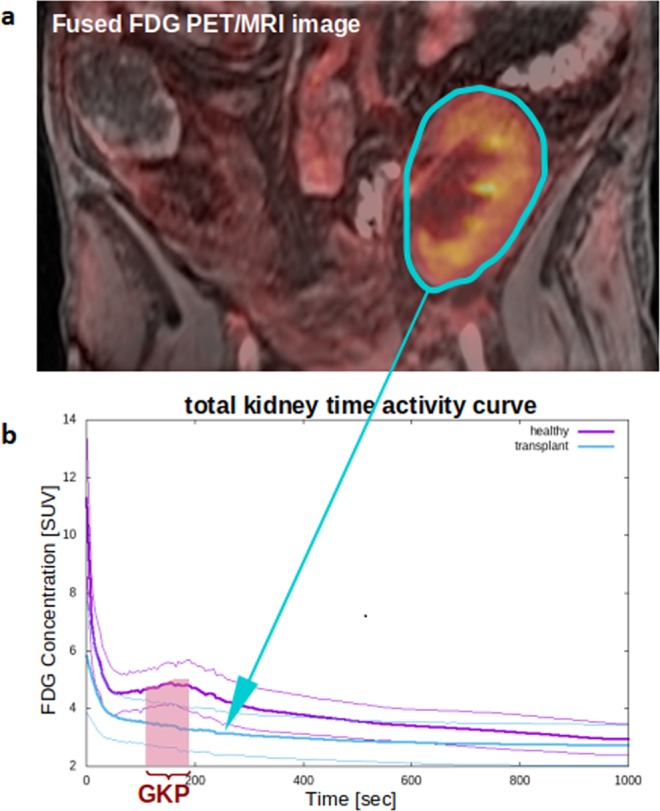
Fused positron emission tomography and magnet resonance image (PET/MRI). (**a**) A delineated volume of interest (VOI) of the total kidneys is schematically indicated in blue. (**b**) FDG time activity curves in units of standardized uptake value (SUV). The curves show the average over all 13 measured transplant kidneys (bold blue line) ± one standard deviation (thin blue lines), and for comparison the average over 48 healthy kidneys (bold purple line) ± one standard deviation (thin purple lines). The general kidney performance (GKP) represents the FDG uptake between minute 2 and 3.

The MRI images were used to delineate volumes-of-interest (VOIs): (1) aorta descendens (below the arteria renalis), drawn by hand in several layers (2) left kidney, (3) right kidney, (4) left kidney cortex (5) right kidney cortex, (6) left kidney medulla and (7) right kidney medulla. VOIs (2–3) were carefully drawn by hand in each layer; all other VOIs were delineated randomly in about 30% of all layers by a threshold VOI selection tool. After image fusion, the FDG concentrations in the according VOIs were measured in units of standardized uptake values (SUV); the time activity curves (TACs), reflecting the tracer concentration over time, were exported for further analysis. In Fig. 1, a fused PET/MRI image of a typical transplant kidney with the corresponding TACs of transplant and healthy control kidneys are presented. FDG TAC analysis was performed using an in-house Java-based tool (programmed with openjdk version 1.8.0_162), for which the aorta input function (AIF) along with the TACs were used as inputs. TACs were smoothed with a Bezier filter and the AIF was fitted with a tri-exponential function starting from its peak.

### Renal function parameters from dynamic scans

To evaluate renal processes, the initial flow (IF) was determined, which is a measure used to assess the renal blood flow. IF is acquired by dividing the maximum measured tracer concentration by the total collected tracer amount in the kidney within the first 60 seconds and can be used to assess the effective renal plasma flow^40^. Additionally, the renal response function (RF) was calculated in order to obtain the collected net tracer concentration over the first minute. The calculation was performed via a deconvolutional analysis as commonly applied on renogram curves from renal scintigraphies. The AIF and the total kidney TAC, from which a response function is obtained, reflect the net tracer concentration in the kidneys^43,44^.

Furthermore, the total renal tracer uptake within the second and third minute of tracer injection was used to quantify the general kidney performance (GKP), see also Fig. 1. The uptake during this interval is commonly used to determine various kidney parameters from renal scintigraphies, e.g. glomerular filtration, renal plasma flow or renal split function^45–47^. Recently, it has been published that this parameter is also affected after sodium-glucose linked transporter-2 inhibitor therapy in patients with type 2 diabetes mellitus^48^.

### Statistical analysis

Statistical analysis was performed with Gnumeric (open source software, version 1.12.20) and LibreOffice Calculator (open source software, version 4.3.7.2). Correlations were calculated with Pearson’s correlation coefficient r, from which a corresponding p value was derived. The significance of the differences between groups, as well as between healthy and transplant kidneys was assessed by the student’s t test with p < 0.05 considered as a statistically significant difference.

## Results

A total of 13 renal transplant recipients were recruited and followed for a minimum of 6 weeks after the PET/MRI scan. Baseline characteristics, underlying renal disease and comorbidities are summarized in Table 1. Eight out of 13 patients were male (61.5%) and the mean age of patients was 57.9 ± 16.4 years.

**Table 1:** Baseline characteristics of the 13 included patients.

ID	Age	Sex	BMI	Bsl sCr [mg/dl]	Underlying renal disease	Comorbidities
1	70	f	23.88	2.8–3.5	Recurrent pyelonephritis	DM II, aortic sclerosis
2	50	m	24.73	2.1–2.8	Refluxnephropathy	HTN, CABG, St.p. Hepatitis C
3	62	f	23.72	2–2.5	Chronic Interstitial nephritis	HIV, St.p. Hepatitis B + E, St.p. thyroid cancer
4	77	m	27.78	2–2.5	Cystic kidney disease	Diverticulosis, sigma adenoma
5	22	m	26.59	2–2.5	Congenital hydronephrosis	HTN, neurogenic bladder
6	58	f	13.98	1.3–3.0	Unknown	Anorexia, chronic pancreatitis, PTX + AutoTX
7	56	m	23.88	3.2–3.5	ADPKD	HTN, liver cysts
8	73	f	38.67	2.5–3	Goodpasture Syndrom	HTN, St.p. PE, adipositas, cholecystolithiasis
9	24	f	25.22	2.5–3	atypical HUS	HTN, St.p. CPR
10	69	f	21.88	0.8–1.2	ADPKD	HTN, Sigma diverticulosis
11	61	m	21.18	2.4–3	ADPKD	COPD, cerebellum stroke
12	66	m	31.02	3–4.0	hepatorenal syndrome	HTN, DM II, LTX, AFIB, adipositas
13	64	m	24.86	3–3.5	FSGS	HTN, DVT, adeno carcinoma of abdomen

Bsl sCr, baseline serum creatinine; ADPKD, autosomal dominant polycystic kidney disease; HUS, hemolytic uremic syndrome; FSGS, focal segmental glomerulosclerosis; DM II, diabetes mellitus type 2; HTN, hypertension; CABG, coronary artery bypass graft; PTX, pancreas transplantation; TX, transplantation; PE, pulmonary embolism; CPR, cardiopulmonary resuscitation; COPD, chronic obstructive pulmonary disease; LTX, liver transplantation; AFIB, atrial fibrillation; DVT, deep venous thrombosis.

With regard to kidney transplantation, information on the sex and age of the graft, data on immunological risk and mismatch are listed in Table 2. Nine patients were enrolled within the first 90 days after transplantation and measured by PET/MRI. The corresponding serum creatinine levels at the time of PET/MRI are shown in Table 2. Twelve patients have had a kidney biopsy before or after the PET/MRI was performed. In one patient no recent biopsy was available. Reasons for kidney biopsies were delayed graft function in 6 cases. A summary on the renal histology is presented in Table 2.

**Table 2:** Information on kidney transplantation, time frame of PET/MRI and biopsy.

ID	Sex kidney	Age kidney	Immunological risk	Mismatch	PET/MRI post TX in days	sCr [mg/dl] PET/MRI	reasons for BX	BX histology
1	f	74	normal	0–1–1	262	4,91	AKI	IFTA
2	f	64	high (DSA+, Luminex+)	1–0–0	10	5,78	DGF	ABMR, tubular injury
3	f	64	high (DSA+, Luminex+)	1–2–1	18	4,75	DGF	no rejection, TMA
4	m	79	normal	0–2–0	6	8,50	DGF	BANFF IIB
5	f	57	normal	1–1–1	12	1,94	AKI	no rejection
6	m	61	normal	2–2–1	89	1,98	AKI	no rejection
7	m	57	normal	1–1–2	66	3,64	DGF	ischemic tubulur injury
8	f	73	high (DSA+, Luminex+)	2–1–2	1734	2,82	na	na
9	m	40	normal	1–2–1	42	2	DGF	no rejection, tubular injury
10	f	60	normal	0–0–0	11	1,07	AKI	no rejection
11	f	60	normal	0–1–1	21	5,14	DGF	no rejection, tubular injury
12	m	65	normal	na	1471	3,03	AKI	glomerulopathy
13	na	na	normal	1–1–0	34904	7	AKI	arteriosclerosis, IFTA

TX, transplantation; sCr, serum creatinine; DSA, donor specific antibody; BX, biopsy; DGF, delayed graft function; AKI, acute kidney injury; TMA, thrombotic microangiopathy; IFTA, interstitial fibrosis and tubulur atrophy; ABMR, antibody mediated rejection; na, not available.

### Recovery

Ten patients had an eGFR <30 ml/min/1.73 m² at the day of the PET/MRI examination, 3 patients presented with an eGFR > 30 ml/min/1.73 m². Patients were treated for DGF, rejection and AKI, accordingly. Six patients showed a positive recovery, i.e. the serum creatinine decreased by > 10% (average: -36%, from 5.1 mg/dl to 3.1 mg/dl) ten days after PET/MRI examination; 4 patients had a negative recovery, i.e. an increased serum creatinine by in average 55% (from 2.0 mg/dl to 3.4 mg/dl); 3 patients showed no or minimal changes between ± 10% (average: -5%, from 3.9 mg/dl to 3.7 mg/dl) in serum creatinine.

### GKP general kidney performance

As depicted in Fig. 1a a VOI was drawn around the kidney after fusion of PET and MRI for calculating the distribution of FDG over time. The mean GKP of the kidney allografts and the healthy kidneys are shown in Fig. 1b. Comparing the GKP in transplanted and healthy kidneys, higher GKP was observed in healthy controls (4.5 ± 1.2 versus 5.6 ± 0.8, respectively), which was significant for the entire kidney (p = 0.0002) and the medulla (p = 0.0004), but no difference was found between the GKP of the cortex (p = 0.59). A difference between the total GKP in patients with low tubular damage (0 to 2) and patients with high tubular damage (3–5) of 22% was found, but this did not reach significance (4.1 versus 3.3, p = 0.17). Also in medulla GKP a non-significant difference of 29% (4.8 versus 3.6, p = 0.07) was seen.

### IF initial flow

The initial flow showed a strong correlation with the change in serum creatinine 10 days after the PET/MRI (r = 0.80, p = 0.001). The averaged IF was lower in patients with positive recovery and stable kidney function (1.5 min^−1^) and higher in patients with negative recovery (1.7 min^−1^), but this difference was not statically significant (p = 0.051).

### RF response function

As described earlier the renal response function (RF) indicates the net amount of FDG within the first minute. The average RF in healthy kidneys did not differ from the average RF in patients after kidney transplantation (p = 0.9). However, patients with severe tubular damage had lower RF (p = 0.0004) whereas patients with no or only mild tubular damage had a significantly higher RF (p < 0.0001).

**Figure 2:**
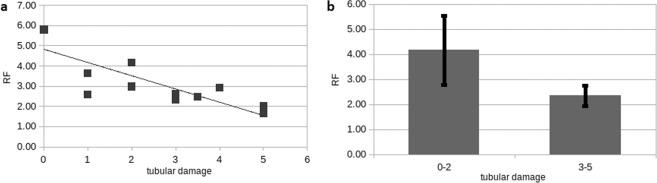
Response function (RF) according to tubular injury. (**a**) Negative correlation between RF and tubular injury (r = −0.83, p = 0.004). (**b**) high RF was associated with mild tubular injury and low RF with severe tubular injury (p = 0.01).

Upon closer examination, a negative correlation between RF and tubular damage was found within the transplant cohort (r = −0.74, p = 0.004) **(**Fig. 2a**)**. Transplanted kidneys with a higher degree of tubular damage showed lower RF compared to no or only slight tubular damage according to renal histology (p = 0.006) **(**Fig. 2b**)**.

With regard to the prognosis of renal recovery 10 days after PET/MRI, a lower RF was associated with a decrease in serum creatinine, but was not significant (2.7 versus 4.3, p = 0.07). Regarding GKP, IF and RF, no influence of the donor’s age, sex or time point of transplantation could be found.

## Discussion

In this prospective observational study 13 patients after KTX with kidney injury underwent radio imaging based on a PET using 2-deoxy-2[18 F] fluoro-D-glucose combined with 3-Tesla MRI. Accordingly, a kinetic model based on the tracer distribution was assessed and high quality morphology on the kidney was used for anatomical allocation. This data was then compared to 24 healthy subjects undergoing the same PET/MRI imaging protocol.

One major finding was a significantly higher GKP in healthy subjects compared to the transplant patients. Interestingly, the medulla and total kidney GKP were significantly higher, whilst the purely cortical GKP showed no difference between the cohorts. This finding indicates that FDG as a glucose analogue is less accumulated in the proximal tubule or even glomeruli, which is also supported by the fact that RF, a parameter which can be understood as measure of the blood entering the kidneys within the first minute, was also higher in patients with a positive recovery. Novel data has previously demonstrated that the Henle’s loop and the capillary system are involved in AKI and its potential recovery, which is supporting our present findings^49–51^.

When looked at the initial flow, a predictive value for serum creatinine development within 10 days after PET/MRI could be seen. Additionally, RF seems of high relevance when classifying tubular damage, as significantly higher RF values were observed in patients with no or mild tubular damage. Although also medulla and total kidney GKP were higher in patients with mild tubular damage, these results were not significant.

In the early phase of kidney transplantation many risk factors due to graft characteristic, warm and cold ischemia time and immunological risk can lead to delayed graft function as a subtype of acute kidney injury. Until now, clinical decisions are based on standard care procedures including close laboratory screening, adequate immunosuppression, avoidance of nephrotoxic medication and maintaining optimal fluid balance^52,53^. However, no commercially accessible tools are available for prediction of DGF and subsequently no estimation on prognosis can be made. In recent years biomarker- guided diagnostic approaches for renal impairment have gained importance. In respect of KTX patients, well investigated markers such as IL-18, NGAL, IGFBP7 and TIMP-2 have shown an association with DGF and worse transplant outcome^54,55^.

Non- invasive radio imaging of renal allograft allows assessment of morphology, perfusion and urologic abnormalities. However, few data regarding functional measurements exist and are yet not implicated in clinical routine. The gold standard for diagnosis of glomerulopathies, tubular damage, interstitial fibrosis and allograft rejection remains the histopathology.

A study performing T1 mapping with MRI in patients early after kidney and lung transplantation showed that the renal cortical relaxation time is longer in patients after kidney transplantation compared to healthy controls^56^. One study has shown a predictive value for allograft dysfunction and fibrosis using a multiparametric 1,5 Tesla magnetic resonance imaging with specific diffusion weighted- imaging and T1 sequences^57^. Another study using functional MRI to assess interstitial fibrosis by arterial spin labeling and apparent diffusion coefficient was able to discriminate between <50% and >50% fibrosis accordingly to histopathology^58^. Clinical trials for ultrasound and MRI have attempted to diagnose acute rejection, but have not been able to discriminate accurately between different pathologies. Imaging protocols with e.g. specific nano-sized particles as contrast agents to identify immune cells as macrophages and T cells have been established, but yet are in experimental stages and only been investigated in murine models^59^.

In a recently published paper the use of FDG PET combined with CT in kidney transplant recipients managed to distinguish between stable graft function and the diagnosis of subclinical rejection in the early phase of transplantation. The level of inflammation proved in allograft biopsies seemed to correlate with the FDG uptake and together with the urinary expression of chemokine CXCL9 and urine creatinine a high negative predictive value on subclinical rejection could be demonstrated. However, these results need to be confirmed in further clinical studies^60^.

To date, no kinetic model in order to evaluate subunits of the kidney and the capillary system has been verified. Therefore, we tried a novel approach based on FDG PET and MRI to not only look at the morphology but shed light to a functional aspect of allograft kidneys.

In the present study we found a potential non- invasive diagnostic tool for prediction of kidney repair according to GKP, IF and RF measured by FDG PET/MRI. The repair mechanism at the renal site, in particular at the proximal tubules requiring glucose as energy supply might be of relevance. Higher FDG uptake might translate into higher energy turnover and cellular repair mechanisms indicating regain of kidney function. However, more prospective studies are required to confirm these findings and evaluate its relevance.

## Conclusion

Dynamic FDG PET/MRI parameters showed a positive correlation with serum creatinine development within 10 days in kidney transplant recipients after AKI and DGF.

### Limitation

No partial volume or motion corrections have been applied on the obtained VOIs. While these effects are insignificant in total kidney VOIs^40^, they are certainly not in small VOIs, mainly the aorta and the medulla, which leads to higher errors. Therefore, the RF was not calculated for the medulla or the cortex VOIs. Although even the GKP from the uncorrected medulla VOI delivered better results, total kidney VOIs might be sufficient to study GKP or RF.

Furthermore, a better parameter to determine initial renal processes would certainly be the rate constant K_1_ from an applied kinetic model. The non-negligible partial volume and motion effects hamper the application of a kinetic model, thus IF was chosen as a rough estimate.

We did not compare the level of total inflammation (ti) as stated in histology with the FDG uptake due to limited number of patients baring a ti score >1. Another limitation of this study is the small cohort size and further studies are needed to validate these results.

## Data Availability

Data, material and associated protocols supporting the findings of this study are available upon request from the corresponding author.
